# The effect of cognitive avoidance on rumination in college students: the chain mediating role of perfectionism and stress

**DOI:** 10.3389/fpsyg.2025.1562927

**Published:** 2025-07-30

**Authors:** Debao An, Jiale Wang, Yeling Xia, Wenlong Xing

**Affiliations:** ^1^Xinjiang University Mental Health Education and Counseling Center, Ürümqi, China; ^2^Wenzhou University School of Education, Ürümqi, China; ^3^School of Physical Education, Xinjiang Normal University, Ürümqi, China; ^4^Xinjiang Mental Health Center (Urumqi Fourth People's Hospital), Ürümqi, China; ^5^Rehabilitation Medicine Center, First Affiliated Hospital of Shihezi University, Shihezi, China

**Keywords:** cognitive avoidance, perfectionism, stress, rumination, chain mediation

## Abstract

This study aimed to examine the effect of cognitive avoidance on rumination among college students, and to explore the mediating roles of perfectionism and stress. Cognitive avoidance involves efforts to evade distressing thoughts, while rumination refers to repetitive negative thinking. A cross-sectional survey was conducted via convenience sampling among students from four universities in Xinjiang, Henan, and Guangdong. A total of 6,000 electronic questionnaires were distributed, and 5,412 valid responses were retained (effective rate: 90.20%). Participants completed the Cognitive Avoidance Questionnaire, Rumination Scale, Positive and Negative Perfectionism Scale, and the Depression Anxiety and Stress Scale (DASS-21). Pearson correlations and mediation analyses using PROCESS were performed. Results showed that cognitive avoidance, perfectionism, stress, and rumination were significantly positively correlated (r = 0.324–0.484, *p* < 0.001). Cognitive avoidance significantly predicted rumination (t = 0.347, *p* < 0.001). Further, three indirect paths were identified: the mediating effect of perfectionism, the mediating effect of stress, and a chain mediating effect through both (95% CIs excluded zero). These findings suggest that cognitive avoidance influences rumination directly and indirectly through perfectionism and stress, offering insights into maladaptive cognitive-emotional patterns in college students.

## 1 Introduction

Individuals often dwell on the causes, processes, and outcomes of negative life events, such as setbacks, exam failures, emotional distress, or workplace difficulties, leading to prolonged and repetitive thinking about the same events. This tendency exacerbates negative emotions and cognitive biases, which can amplify psychological distress and, in severe cases, contribute to depression and other mental health issues (Liu et al., 2024). This phenomenon is referred to as “Rumination,” a concept introduced by Nolen-Hoeksema in her research on depression. Nolen-Hoeksema (1991) defined rumination as the passive and repetitive focus on one's negative emotions and their causes, consequences, and implications following adverse life events. Research has consistently demonstrated a strong link between negative rumination and various mental disorders, including depression, anxiety, bipolar disorder, and post-traumatic stress disorder (Constantin et al., 2017; Kertz et al., 2019; Murray et al., 2019; Mihailova and Jobson, 2020; Wang et al., 2025). However, not all rumination is inherently negative. Studies by Johnson and McKenzie et al. found that while patients with depression and bipolar disorder both exhibit high levels of negative rumination, bipolar disorder patients also show a propensity for positive rumination (Johnson et al., 2008). Feldman et al. further explored the concept of positive rumination in their studies, and in China, Wang (2016) developed the Chinese College Student Rumination Scale, which includes dimensions for both positive and negative rumination.

Most domestic and international studies have focused on the effects of rumination on individuals, with relatively little exploration of the underlying causes. Not all individuals experiencing negative sensations, thoughts, memories, or emotions engage in rumination or deep cognitive processing. Some individuals adopt avoidance strategies, such as experiential avoidance and cognitive avoidance. Studies have shown that experiential avoidance positively predicts rumination (Yang et al., 2021). In daily life, intrusive images or thoughts that flash through one's mind are often deliberately or unintentionally suppressed or avoided to prevent disruption of ongoing activities. Strategies such as thought suppression, attention diversion, and thought substitution fall under the phenomenon known as “Cognitive Avoidance” (Behar et al., 2009). Based on this, the present study hypothesizes that H1: Cognitive avoidance is closely related to rumination and positively predicts it.

**Figure 1:**
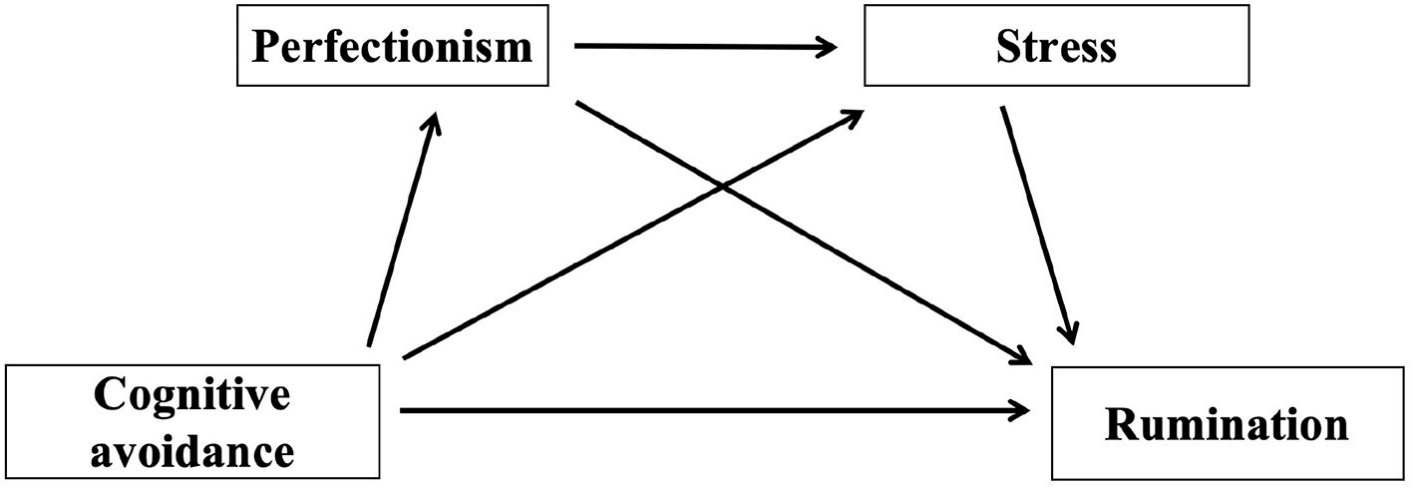
Hypothesized a mediation model.

Research has also shown that perfectionism positively predicts rumination, with individuals exhibiting higher levels of perfectionism engaging in deeper rumination (Chen et al., 2022; Flett et al., 2016). Zhang D. et al. (2021) found that stress in college students positively predicts rumination and indirectly affects sleep quality through rumination (Wang et al., 2025). Similarly, Xie (2021) found that perfectionism in corporate employees positively affects work stress, which in turn indirectly influences work addiction. Avoiding intrusive thoughts or images in daily life, whether consciously or unconsciously, to maintain focus on current tasks, is also a manifestation of perfectionism, reflecting the pursuit of an ideal or flawless personality trait (Chen et al., 2015; Wang et al., 2025). Based on these findings, the present study hypothesizes that H2: Perfectionism and stress serve as chain mediators in the relationship between cognitive avoidance and rumination. Consequently, a hypothetical path model has been constructed (Figure 1).

## 2 Methods

### 2.1 Participants

This study employed a convenience sampling method to conduct a classroom-based cross-sectional survey among university students from four higher education institutions in Xinjiang, Henan, and Guangdong provinces. Before distributing the questionnaire, researchers provided participants with detailed information about the study's purpose, main content, confidentiality of data, and its intended use. The survey was conducted using an electronic questionnaire. The first page of the questionnaire included an informed consent form, which participants had to read and agree to before proceeding. Those who declined were directed out of the questionnaire interface. Participation in the survey was entirely anonymous and voluntary, with an average completion time of 20 min.

The study received approval from the Medical Ethics Committee of the authors' institution prior to its implementation. A total of 6,000 questionnaires were distributed. After excluding invalid responses, 5,412 valid questionnaires were retained, resulting in an effective response rate of 90.20%. Invalid responses included questionnaires with patterned answers or excessively short or long response times.

Among the valid respondents, participants ranged in age from 16 to 30 years, with an average age of 19.89 years (SD = 2.08). The sample included 2,803 males (51.79%) and 2,609 females (48.21%). Regarding residential background, 2,060 participants (38.06%) were from rural areas, 1,052 (19.44%) from towns, and 2,300 (42.50%) from urban areas.

### 2.2 Measures

#### 2.2.1 Cognitive avoidance

The Cognitive Avoidance Questionnaire (CAQ), introduced and revised by Jiao et al. (2017), was used in this study. The CAQ comprises five factors and 25 items, measuring the following dimensions: thought suppression, thought substitution, attention diversion, avoidance of threatening stimuli, and imagery conversion. Responses are scored on a 5-point Likert scale ranging from “not at all typical” (1) to “completely typical” (5). The reliability and validity of the CAQ were confirmed by Jiao et al., with a Cronbach's alpha coefficient of 0.930 for the overall scale and coefficients ranging from 0.732 to 0.839 for the subscales. In this study, the internal consistency of the CAQ yielded a Cronbach's alpha of 0.952, indicating excellent reliability and suitability for further analysis.

#### 2.2.2 Rumination

The Rumination Scale, developed by Wang (2016), was employed to assess rumination among university students. This scale includes two dimensions—positive rumination and negative rumination—comprising a total of 23 items. Responses are rated on a 4-point Likert scale, ranging from “never” (1) to “always” (4). The scale demonstrated an internal consistency reliability coefficient of 0.740 in its original development. In this study, the internal consistency of the scale produced a Cronbach's alpha coefficient of 0.799, indicating good reliability and appropriateness for further analysis.

#### 2.2.3 Perfectionism

The Positive and Negative Perfectionism Scale (PANPS), originally developed by Terry-Short et al. (1995) and translated and revised by Zhou (2012), was utilized in this study (Zhou, 2012). The revised scale consists of 25 items across two dimensions: positive perfectionism and negative perfectionism. Responses are measured using a 5-point Likert scale, ranging from “strongly disagree” (1) to “strongly agree” (5). Higher mean scores in each dimension indicate stronger tendencies toward positive or negative perfectionism, respectively. The original scale reported an internal consistency coefficient of 0.84. In this study, the PANPS achieved a Cronbach's alpha coefficient of 0.916, confirming excellent reliability and its suitability for further analysis.

#### 2.2.4 Stress

The stress subscale of the 21-Item Depression Anxiety and Stress Scale (DASS-21), Simplified Chinese version, was employed to measure participants' stress levels (Gong et al., 2010; Lu et al., 2020). This subscale uses a 4-point Likert scale ranging from “does not apply” (0) to “applies very much” (3). In this study, the internal consistency of the stress subscale yielded a Cronbach's alpha coefficient of 0.861, indicating good reliability and appropriateness for further analysis.

### 2.3 Data processing and analysis

Statistical analyses were conducted using SPSS 26.0. Initially, a common method bias test was performed, with a threshold of < 40% indicating no significant common method bias. Next, we conducted a correlation analysis of the demographic characteristics and key variables of the study participants. Prior to further analysis, the data for the key variables were standardized. To test the proposed hypotheses, the relationship between cognitive avoidance and rumination was examined using the PROCESS macro for SPSS (Model 6). The mediating roles of perfectionism and stress were also explored. A bootstrap resampling procedure with 5,000 iterations was used to assess model fit and estimate the 95% confidence intervals (95% CI), ensuring robustness in the analysis. Demographic variables were included as covariates to control for their potential effects. The significance level was set at 0.05.

## 3 Results

### 3.1 Common method bias test

The results of the common method bias test identified 12 factors with eigenvalues >1, with the first factor accounting for 17.89% of the total variance, which is below the 40% threshold. This indicates that there is no significant risk of common method bias in this study.

### 3.2 Relationships among cognitive avoidance, rumination, perfectionism, and stress

Pearson's correlation analysis was used to examine the relationships among cognitive avoidance, rumination, perfectionism, and stress in university students. The results revealed that cognitive avoidance was significantly positively correlated with rumination (r = 0.347, *P* < 0.001), perfectionism (r = 0.324, *P* < 0.001), and stress (r = 0.484, *P* < 0.001). Rumination was significantly positively correlated with perfectionism (r = 0.471, *P* < 0.001) and stress (r = 0.324, *P* < 0.001). However, the positive perfectionism dimension of perfectionism showed no significant correlation with the thought substitution dimension of cognitive avoidance (*P* > 0.05) or with stress (*P* > 0.05). Detailed results are presented in Table 1.

**Table 1:** Correlation matrix of cognitive avoidance, rumination, perfectionism, and stress.

**Variables**	**1**	**2**	**3**	**4**	**5**	**6**	**7**	**8**	**9**	**10**	**11**
1. Thought substitution	1										
2. Imagery-based thinking	0.704^***^	1									
3. Attention shifting	0.649^***^	0.717^***^	1								
4. Avoidance of dangerous stimuli	0.689^***^	0.724^***^	0.755^***^	1							
5. Thought suppression	0.600^***^	0.659^***^	0.718^***^	0.764^***^	1						
6. Cognitive avoidance	0.826^***^	0.873^***^	0.885^***^	0.907^***^	0.862^***^	1					
7.· Rumination	0.278^***^	0.331^***^	0.301^***^	0.307^***^	0.292^***^	0.347^***^	1				
8. Positive perfectionism	0.005	0.143^***^	0.168^***^	0.138^***^	0.147^***^	0.142^***^	0.393^***^	1			
9. Negative perfectionism	0.373^***^	0.380^***^	0.367^***^	0.402^***^	0.383^***^	0.437^***^	0.418^***^	0.478^***^	1		
10. Perfectionism	0.204^***^	0.295^***^	0.303^***^	0.303^***^	0.298^***^	0.324^***^	0.471^***^	0.868^***^	0.845^***^	1	
11. Stress	0.465^***^	0.415^***^	0.375^***^	0.447^***^	0.416^***^	0.484^***^	0.324^***^	−0.003	0.455^***^	0.244^***^	1

^*^p < 0.05; ^**^p < 0.01; ^***^p < 0.001

### 3.3 Mediating effects among key variables

The mediation analysis followed the procedure outlined by Wen and Ye (2014). Before the analysis, all variables were standardized into Z-scores. Controlling for age and gender, we first examined the predictive effects of cognitive avoidance, perfectionism, and stress on rumination, with rumination as the dependent variable. The results (Table 2) revealed that cognitive avoidance significantly and positively predicted rumination (t = 0.347, *P* < 0.001), perfectionism significantly and positively predicted rumination (t = 0.403, *P* < 0.001), and stress significantly and positively predicted rumination (t = 0.158, *P* < 0.001).

**Table 2:** Regression analysis with rumination as the outcome variable.

**Variables**	**Step 1**		**Step 2**		**Step 3**		**Step 4**	
	β	***t***	β	***t***	β	***t***	β	***t***
Age	0.000	0.012	−0.012	−0.961	0.021	1.808	0.012	1.047
Gender	−0.023	−1.662	−0.028	−2.192^*^	−0.036	−3.106^*^	−0.033	−2.852^**^
Cognitive avoidance			0.347	27.236^***^	0.216	17.460^***^	0.145	10.661^***^
Perfectionism					0.403	32.587^***^	0.387	31.487^***^
Stress							0.158	11.904^***^
*R^2^*	0.001		0.121		0.265		0.283	
*F*	1.381		248.315^***^		488.257^***^		429.111^***^	

^*^p < 0.05; ^**^p < 0.01; ^***^p < 0.001. Step 1 includes control variables (age and gender); Step 2 adds cognitive avoidance; Step 3 adds perfectionism; Step 4 adds stress. This stepwise regression approach was used to test the incremental explanatory power of each variable on rumination.

Next, we tested the predictive effects of cognitive avoidance and perfectionism on stress. The results (Table 3) showed that cognitive avoidance significantly and positively predicted stress (t = 0.483, *P* < 0.001), and perfectionism also significantly and positively predicted stress (t = 0.103, *P* < 0.001). These findings indicate that perfectionism not only directly affects rumination but also indirectly affects it via stress. Specifically, stress partially mediates the relationship between perfectionism and rumination. Combining the results in Table 2, we found that cognitive avoidance not only directly affects rumination but also indirectly affects it through stress, indicating that stress partially mediates the relationship between cognitive avoidance and rumination.

**Table 3:** Regression analysis with stress as the outcome variable.

**Variables**	**Step 1**		**Step 2**		**Step 3**	
	β	**t**	β	**t**	β	**t**
Age	0.066	4.869	0.049	4.106^***^	0.057	4.833^***^
Gender	−0.012	−0.877	−0.019	−1.629	−0.021	−1.817
Cognitive avoidance			0.483	40.643^***^	0.449	35.945^***^
Perfectionism					0.103	8.196^***^
*R^2^*	0.004		0.237		0.245	
*F*	12.297^***^		561.307^***^		442.925^***^	

^*^p < 0.05; ^**^p < 0.01; ^***^p < 0.001. Step 1 includes control variables; Step 2 adds cognitive avoidance; Step 3 adds perfectionism.

Finally, we tested the predictive effect of cognitive avoidance on perfectionism. The results (Table 4) indicated that cognitive avoidance significantly and positively predicted perfectionism (t = 0.327, *P* < 0.001). Together with the findings in Table 3, it was observed that cognitive avoidance not only directly affects stress but also indirectly affects it via perfectionism, suggesting that perfectionism mediates the relationship between cognitive avoidance and stress. From Table 2, we further found that cognitive avoidance not only directly affects rumination but also indirectly affects it via perfectionism. The full mediation model is illustrated in Figure 1.

**Table 4:** Regression analysis with perfectionism as the outcome variable.

**Variables**	**Step 1**		**Step 2**	
	β	**t**	β	**t**
Age	−0.071	−5.252^***^	−0.083	−6.468^***^
Gender	0.026	1.886	0.021	1.602
Cognitive avoidance			0.327	25.516^***^
R^2^	0.005		0.112	
F	15.702^***^		228.740^***^	

^*^p < 0.05; ^**^p < 0.01; ^***^p < 0.001. Step 1 includes control variables; Step 2 adds cognitive avoidance.

### 3.4 Indirect and chain mediating effects of perfectionism and stress

**Figure 2:**
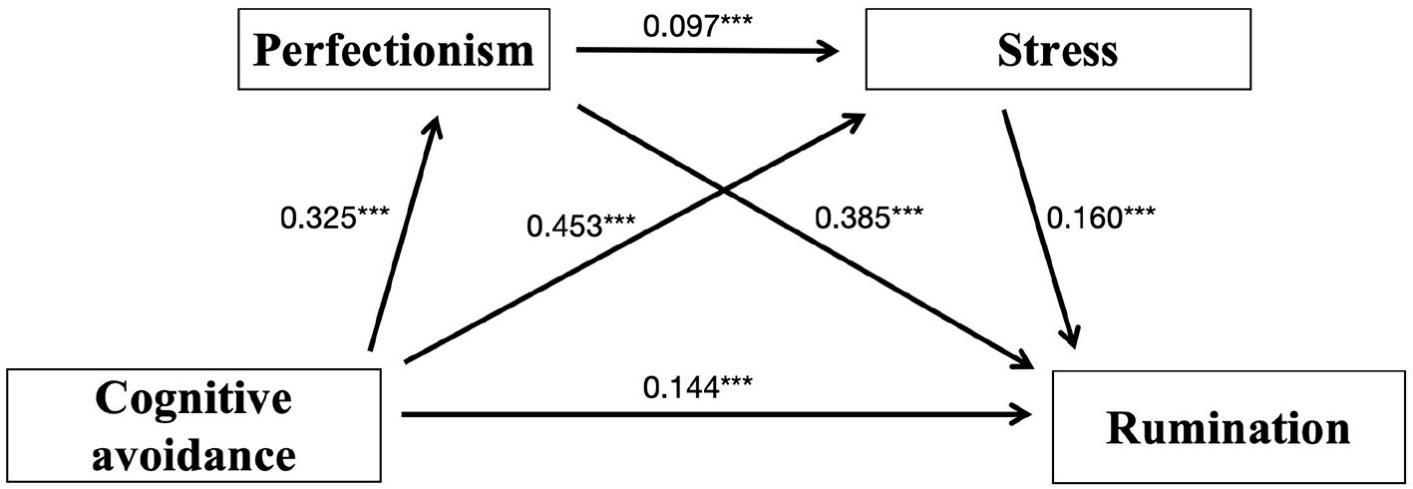
Chain mediation model diagram. *p < 0.05, **p < 0.01, ***p < 0.001.

Using cognitive avoidance as the predictor variable, perfectionism and stress as the mediating variables, and rumination as the dependent variable, we employed the bias-corrected percentile bootstrap method to analyze mediation effects. The analysis involved 5,000 resampling iterations and calculated 95% confidence intervals. The results indicated that the chain mediation effect of perfectionism and stress was significant, as the confidence interval did not include zero ([0.0034, 0.0070], *P* < 0.001). These findings further confirm the chain mediation effect of perfectionism and stress between cognitive avoidance and rumination. The direct effect of cognitive avoidance on rumination accounted for 41.65% of the total effect, while the indirect effect through perfectionism and stress accounted for 58.35%. Among the indirect effects, the mediation effect of perfectionism accounted for 36.03%, the mediation effect of stress accounted for 20.88%, and the chain mediation effect of perfectionism and stress accounted for 1.44%. Detailed mediation effects and pathways are presented in Table 5 and Figure 2.

**Table 5:** Mediation model path analysis.

**Effect**	**Path relationship**	**95% Confidence interval**	**Effect value**	**Effect size**
Direct effect	Cognitive avoidance → Rumination	[0.1178,0.1710]	0.1444	41.65%
Indirect effect	Cognitive avoidance → Perfectionism → Rumination	[0.1096,0.1429]	0.1249	36.03%
	Cognitive avoidance → Stress → Rumination	[0.0584,0.0878]	0.0724	20.88%
	Cognitive avoidance → Perfectionism → Stress → Rumination	[0.0034,0.0070]	0.0050	1.44%
Total mediation effect			0.2023	58.35%
Total effect			0.3467	100%

^*^p < 0.05; ^**^p < 0.01; ^***^p < 0.001.

## 4 Discussion

Cognitive avoidance is a significant predictor of rumination. Our study found that cognitive avoidance positively influences rumination, with higher levels of cognitive avoidance associated with more pronounced rumination. This finding supports hypothesis H1. Previous research by Yang et al. (2021) demonstrated a significant positive correlation between experiential avoidance and rumination among clinical nurses. Their regression analysis further confirmed the significant positive predictive effect of experiential avoidance on rumination (Yang et al., 2021). Although both experiential avoidance and cognitive avoidance emphasize evading negative events or intrusive thoughts (e.g., thought suppression), experiential avoidance is broader in scope. It includes the avoidance, alteration, or suppression of thoughts, emotions, memories, and even physical sensations (Hayes et al., 1996). In contrast, cognitive avoidance is more specific and operationally focused, involving deliberate or unintentional avoidance of intrusive thoughts or imagery, such as thought suppression, attentional shifts, or cognitive substitutions (Shen, 2020). Our findings reveal that cognitive avoidance is positively correlated with rumination and can significantly influence it. When university students deliberately or unintentionally evade intrusive thoughts or images that interfere with ongoing activities, they aim to reduce the influence of these negative stimuli. However, this process often leads to the inadvertent processing of such stimuli, triggering rumination.

Our study found that cognitive avoidance influences rumination through the mediating role of perfectionism, accounting for 36.03% of the total mediating effect. Cognitive avoidance during the evasion of intrusive thoughts or images reflects a pursuit of perfection in ongoing activities. Individuals with higher tendencies toward perfectionism are more likely to exhibit heightened levels of rumination (Gan et al., 2017; Chen et al., 2022).

Cognitive avoidance also affects rumination through the mediating role of stress, with stress accounting for 20.88% of the total mediating effect. This finding highlights the crucial role of stress in understanding the impact of cognitive avoidance on rumination. Controlling intrusive thoughts amplifies individuals' perceptions of stress, which, in turn, triggers rumination (Zhang D. et al., 2021). This finding supports the stress-reactivity model of rumination, which posits that individuals exhibit rumination tendencies when faced with stress (Robinson and Alloy, 2003).

Further analysis revealed that perfectionism and stress together form a chain mediating effect between cognitive avoidance and rumination, supporting hypothesis H2. Cognitive avoidance not only directly impacts rumination but also exerts an indirect influence via perfectionism and stress. Individuals attempting to control intrusive thoughts often develop perfectionistic tendencies, which heighten their perceived stress levels (Xie, 2021), ultimately exacerbating rumination. This result suggests that when cognitive avoidance's effects on rumination cannot be effectively mitigated, strategies such as reducing perfectionistic tendencies and alleviating stress may help lower rumination levels. This approach could also minimize the physiological and psychological harm caused by rumination (Huang et al., 2019; Zhang K. et al., 2021; Guo and Wu, 2011; Zhong et al., 2015; Wang et al., 2025).

This study provides novel insights into the relationship between cognitive avoidance and rumination among university students, particularly highlighting the mediating roles of perfectionism and stress—mechanisms that have received limited attention in previous research. However, several limitations should be noted. First, the reliance on self-reported data may introduce subjective biases, such as social desirability or recall errors, potentially affecting the objectivity and reliability of the results. Second, the representativeness of the sample may be limited due to its lack of diversity, which constrains the generalizability and external validity of the findings. Third, the cross-sectional design of this study prevents the establishment of causal relationships among the variables. Future research should employ longitudinal or experimental designs to explore these mechanisms over time. Despite these limitations, the study offers important theoretical and practical implications. Theoretically, it enriches the understanding of how cognitive avoidance contributes to rumination by uncovering the chain mediating effects of perfectionism and stress. Practically, the findings suggest that interventions aimed at reducing rumination among college students should consider addressing cognitive avoidance strategies and maladaptive perfectionistic tendencies, as well as managing perceived stress.

## 5 Conclusion

This study further elucidates the mechanisms through which cognitive avoidance influences rumination among university students, offering valuable theoretical and practical implications for reducing rumination levels and promoting positive psychological traits. By enriching the theoretical framework surrounding rumination, this research provides actionable directions for mental health education among university students. Psychological counseling services in higher education institutions can focus on moderating perfectionism, alleviating academic and work-related stress, and integrating cognitive-behavioral interventions to help students manage negative emotions and reduce the likelihood of rumination. Additionally, strengthening mental health education campaigns to cultivate self-acceptance and adaptive cognitive strategies can foster resilience and contribute to the holistic development of mentally and emotionally healthy university students. These strategies provide a feasible and effective pathway for nurturing a positive and thriving student population.

## Data Availability

The raw data supporting the conclusions of this article will be made available by the authors, without undue reservation.
